# (*E*)-3-(2,6-Dichloro­phen­yl)-1-(4-methoxy­phen­yl)prop-2-en-1-one

**DOI:** 10.1107/S1600536809020145

**Published:** 2009-06-06

**Authors:** Lotfi Benmekhbi, Ratiba Belhouas, Sofiane Bouacida, Salima Mosbah, Leila Bencharif

**Affiliations:** a Département de Chimie et de Chimie Pharmaceutique, Université de Msila, 28000 Algeria; b Faculté de Chimie, USTHB, BP32, El-Alia, Bab-Ezzouar, Alger, Algeria; c Laboratoire de Chimie Moléculaire, du Contrôle de l’Environnement et de Mesures Physico-Chimiques, Faculté des Sciences, Département de Chimie, Université Mentouri, 25000 Constantine, Algeria; d Laboratoire de Chimie des Matériaux, Université de Mentouri, 25000 Constantine, Algeria

## Abstract

In the title compound, C_16_H_12_Cl_2_O_2_, the dichloro­phenyl and methoxy­phenyl groups are linked by a prop-2-en-1-one group. The C=C double bond is *trans* configured. The mol­ecule is not planar, as can be seen from the dihedral angle of 6.21 (7)° between the planes of the two rings. The crystal structure can be described by two types of crossed layers which are parallel to (110) and (1
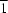
0).

## Related literature

For background to the applications of chalcones, see: Liu *et al.* (2003 ▶); Li *et al.* (1995 ▶); Hsieh *et al.* (1998 ▶); Barford *et al.* (2002 ▶); Rojas *et al.* (2002 ▶); Nerya *et al.* (2006 ▶); Yang *et al.* (2000 ▶); Ducki *et al.* (1998 ▶); Goto *et al.* (1991 ▶); Indira *et al.* (2002 ▶); Lawrence *et al.* (2001 ▶); Nielsen *et al.* (2005 ▶); Sarker & Nahar (2004 ▶); Sarojini *et al.* (2006 ▶). For related structures, see: Yathirajan *et al.* (2007 ▶); Butcher *et al.* (2007 ▶); Fischer *et al.* (2007 ▶).
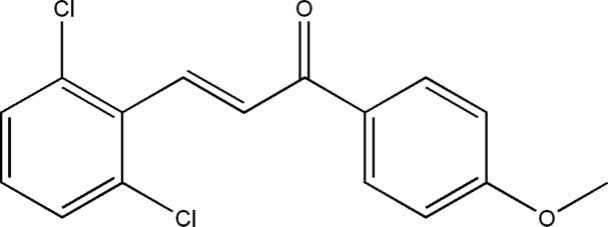


## Experimental

### 

#### Crystal data

C_16_H_12_Cl_2_O_2_
*M* *_r_* = 307.16
Orthorhombic, 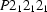

*a* = 6.4793 (2) Å
*b* = 12.9807 (5) Å
*c* = 16.7819 (8) Å
*V* = 1411.46 (10) Å^3^
*Z* = 4
Mo *K*α radiation
μ = 0.46 mm^−1^
*T* = 100 K
0.37 × 0.28 × 0.2 mm

#### Data collection

Bruker APEXII diffractometer
Absorption correction: multi-scan (*SADABS*, Bruker, 1998 ▶) *T*_min_ = 0.824, *T*_max_ = 0.913
6643 measured reflections
3211 independent reflections
2964 reflections with *I* > 2σ(*I*)
*R*_int_ = 0.029

#### Refinement

*R*[*F* ^2^ > 2σ(*F* ^2^)] = 0.036
*wR*(*F* ^2^) = 0.090
*S* = 1.05
3211 reflections
182 parameters
H-atom parameters constrained
Δρ_max_ = 0.51 e Å^−3^
Δρ_min_ = −0.20 e Å^−3^
Absolute structure: Flack (1983 ▶), 1331 Friedel pairs
Flack parameter: 0.01 (6)

### 

Data collection: *APEX2* (Bruker, 2001 ▶); cell refinement: *SAINT* (Bruker, 2001 ▶); data reduction: *SAINT*; program(s) used to solve structure: *SIR2002* (Burla *et al.*, 2003 ▶); program(s) used to refine structure: *SHELXL97* (Sheldrick, 2008 ▶); molecular graphics: *ORTEP-3 for Windows* (Farrugia, 1997 ▶); software used to prepare material for publication: *WinGX* (Farrugia, 1999 ▶) and *DIAMOND* (Brandenburg & Berndt, 2001 ▶).

## Supplementary Material

Crystal structure: contains datablocks global, I. DOI: 10.1107/S1600536809020145/bq2141sup1.cif

Structure factors: contains datablocks I. DOI: 10.1107/S1600536809020145/bq2141Isup2.hkl

Additional supplementary materials:  crystallographic information; 3D view; checkCIF report

## Figures and Tables

**Table 1:** Hydrogen-bond geometry (Å, °)

*D*—H⋯*A*	*D*—H	H⋯*A*	*D*⋯*A*	*D*—H⋯*A*
C4—H4⋯*Cg*1^i^	0.95	2.84	3.727	157
C7—H7⋯*Cg*2^i^	0.95	2.85	3.360	115

Symmetry code: (i) 
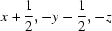
. *Cg*1 and *Cg*2 are the centroids of the C1–C6 and C11–C16 rings, respectively.

**Fig. 1.**
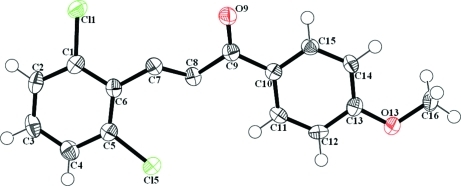
(Farrugia, 1997) The structure of the title compound with the atomic labeling scheme. Displacements are drawn at the 50% probability level.

**Fig. 2.**
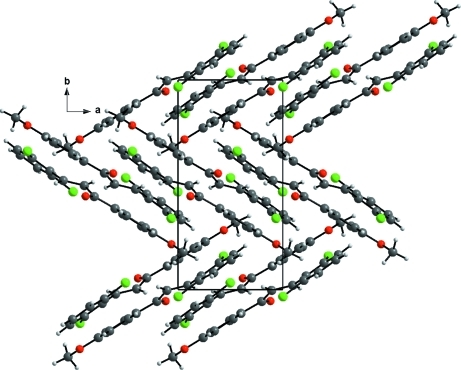
(Brandenburg & Berndt, 2001) A diagram of the layered crystal packing in (I), viewed down the c axis.

## supplementary crystallographic information

### Comment

For a structurally simple group of compounds, chalcones have displayed an impressive array for biological activities, among which anti-malarial (Liu *et al.*, 2003), anti protozoal (Li *et al.*, 1995), anti-inflammatory (Hsieh *et al.*, 1998), immunomodulatory (Barford *et al.*, 2002), nitric oxid inhibition (Rojas *et al.*, 2002), tyronase inhibition (Nerya *et al.*, 2006), cytotoxic (Yang *et al.*, 2000) and anticancer (Ducki *et al.*, 1998) activities have been cited in literature.

Chalcone may be useful for the chemotherapy of leishmanisis among others (Lawrence *et al.*, 2001), they are also used as antibiotics (Nielsen *et al.*, 2005). They were synthesized by a base catalyzed Claisen-Schmidt condensation of aromatic aldehydes and ketones. A natural medicine genus Angelica is known to contain large number of naturally occurring chalcones (Sarker *et al.*, 2004). Chalcone derivatives are recognized for NLO properties and have good crystallization ability (Goto *et al.*, 1991; Indira *et al.*, 2002; Sarojini *et al.*, 2006).

Structure of few related chalcones *viz*., (2E) -1- (2,4-dichlorophenyl) -3-(2-hydrox-3-metoxyphenyl)prop -2-en-1-one (Yathirajan *et al.*, 2007), (2E) -1- (3-hydroxyphenyl) -3-(4-methylphenyl)prop-2-en-1-one (Butcher *et al.*, 2007), (2E)-3-(biphenyl-4-yl)-1-(4-methoxyphenyl)prop-2-en-1-one (Fischer *et al.*, 2007).

The molecular structure of (I), and the atomic numbering used, is illustrated in Fig. 1. A diagram of the layered crystal packing in the unit cell of (I) is shown in Fig. 2. A substituted chalcone adopts an E configuration with respect to the C=C bond of the enone unit. The molecule is not planar, as can be seen from the dihedral angle of 6.21 (7)° between the two rings. The crystal structure can be described by two types of crossed layers, parallel to (110) and (1–10) respectively (Fig. 2).

The packing is stabilized by Van der Walls interactions and by C—H···π interactions resulting in the formation of three dimensional network (Table 1.).

### Experimental

To a mixture of 2,6 dichlorobenzaldehyde (1.75 g, 0.01 mol) and 4-methoxyacetophenone (1.50 g, 0.01 mol) in ethanol 20 ml in the presence of a catalytic amount of sodium hydroxide solution (5 ml) was added slowly with stirring (6 h), the contents of the flask were poured into ice cold water (500 ml) and left to stand for 5 h. The resulting crude solid was filtered and purified by recrystallization in ethanol. Crystal suitable for *x*-ray analysis was grown by slow evaporation of an acetone solution at room temperature.

### Refinement

All H atoms were localized in Fourier maps but introduced in calculated positions and treated as riding on their parent C atoms with C—H = 0.95–0.98Å and *U*_iso_(H) =1.2–1.5(carrier atom).

### Crystal data

**Table:**

C_16_H_12_Cl_2_O_2_	*F*(000) = 632
*M**_r_* = 307.16	*D*_x_ = 1.445 Mg m^−^^3^
Orthorhombic, *P*2_1_2_1_2_1_	Mo *K*α radiation, λ = 0.71073 Å
Hall symbol: P 2ac 2ab	Cell parameters from 3041 reflections
*a* = 6.4793 (2) Å	θ = 2.4–27.4°
*b* = 12.9807 (5) Å	µ = 0.46 mm^−^^1^
*c* = 16.7819 (8) Å	*T* = 100 K
*V* = 1411.46 (10) Å^3^	Prism, colourless
*Z* = 4	0.37 × 0.28 × 0.2 mm

### Data collection

**Table:**

Bruker APEXII diffractometer	2964 reflections with *I* > 2σ(*I*)
graphite	*R*_int_ = 0.029
CCD rotation images, thin slices scans	θ_max_ = 27.4°, θ_min_ = 3.5°
Absorption correction: multi-scan (*SADABS*, Bruker, 1998)	*h* = −6→8
*T*_min_ = 0.824, *T*_max_ = 0.913	*k* = −15→16
6643 measured reflections	*l* = −20→21
3211 independent reflections	

### Refinement

**Table:**

Refinement on *F*^2^	Secondary atom site location: difference Fourier map
Least-squares matrix: full	Hydrogen site location: inferred from neighbouring sites
*R*[*F*^2^ > 2σ(*F*^2^)] = 0.036	H-atom parameters constrained
*wR*(*F*^2^) = 0.090	*w* = 1/[σ^2^(*F*_o_^2^) + (0.0409*P*)^2^ + 0.5074*P*] where *P* = (*F*_o_^2^ + 2*F*_c_^2^)/3
*S* = 1.05	(Δ/σ)_max_ = 0.002
3211 reflections	Δρ_max_ = 0.51 e Å^−^^3^
182 parameters	Δρ_min_ = −0.20 e Å^−^^3^
0 restraints	Absolute structure: Flack (1983), 1331 Friedel pairs
Primary atom site location: structure-invariant direct methods	Flack parameter: 0.01 (6)

### Special details

**Table:**

Geometry. All e.s.d.'s (except the e.s.d. in the dihedral angle between two l.s. planes) are estimated using the full covariance matrix. The cell e.s.d.'s are taken into account individually in the estimation of e.s.d.'s in distances, angles and torsion angles; correlations between e.s.d.'s in cell parameters are only used when they are defined by crystal symmetry. An approximate (isotropic) treatment of cell e.s.d.'s is used for estimating e.s.d.'s involving l.s. planes.
Refinement. Refinement of *F*^2^ against ALL reflections. The weighted *R*-factor *wR* and goodness of fit *S* are based on *F*^2^, conventional *R*-factors *R* are based on *F*, with *F* set to zero for negative *F*^2^. The threshold expression of *F*^2^ > σ(*F*^2^) is used only for calculating *R*-factors(gt) *etc*. and is not relevant to the choice of reflections for refinement. *R*-factors based on *F*^2^ are statistically about twice as large as those based on *F*, and *R*- factors based on ALL data will be even larger.

### Fractional atomic coordinates and isotropic or equivalent isotropic displacement parameters (Å^2^)

**Table:**

	*x*	*y*	*z*	*U*_iso_*/*U*_eq_	
Cl1	0.49869 (7)	1.03293 (4)	0.58897 (3)	0.02481 (12)	
Cl5	0.06450 (7)	0.84015 (4)	0.35072 (3)	0.02267 (12)	
C1	0.2990 (3)	0.95544 (14)	0.55269 (13)	0.0174 (4)	
C2	0.1599 (3)	0.91924 (15)	0.60937 (13)	0.0211 (4)	
H2	0.1779	0.9363	0.664	0.025*	
C3	−0.0051 (3)	0.85821 (14)	0.58604 (13)	0.0226 (4)	
H3	−0.1001	0.8333	0.6246	0.027*	
C4	−0.0312 (3)	0.83360 (14)	0.50613 (12)	0.0206 (4)	
H4	−0.1422	0.7907	0.4898	0.025*	
C5	0.1068 (3)	0.87235 (14)	0.45044 (12)	0.0167 (4)	
C6	0.2774 (3)	0.93459 (13)	0.47063 (13)	0.0149 (4)	
C7	0.4050 (3)	0.97926 (13)	0.40676 (12)	0.0150 (4)	
H7	0.3354	0.9928	0.358	0.018*	
C8	0.6062 (3)	1.00344 (13)	0.40815 (13)	0.0169 (4)	
H8	0.6877	0.9871	0.4535	0.02*	
C9	0.7012 (3)	1.05587 (14)	0.33855 (12)	0.0172 (4)	
O10	0.61836 (19)	1.05435 (10)	0.27255 (9)	0.0215 (3)	
C11	0.8991 (3)	1.11288 (13)	0.35089 (12)	0.0154 (4)	
C12	0.9741 (3)	1.13891 (13)	0.42704 (11)	0.0164 (4)	
H12	0.9017	1.1168	0.4732	0.02*	
C13	1.1527 (3)	1.19652 (14)	0.43510 (12)	0.0172 (4)	
H13	1.2022	1.2139	0.4867	0.021*	
C14	1.0073 (3)	1.14718 (13)	0.28402 (11)	0.0170 (4)	
H14	0.9569	1.131	0.2324	0.02*	
C15	1.1874 (3)	1.20463 (14)	0.29156 (12)	0.0173 (4)	
H15	1.26	1.227	0.2455	0.021*	
C16	1.2603 (3)	1.22906 (14)	0.36746 (12)	0.0172 (4)	
O17	1.4376 (2)	1.28344 (10)	0.38116 (8)	0.0224 (3)	
C18	1.5488 (3)	1.32052 (14)	0.31247 (13)	0.0225 (4)	
H18A	1.5785	1.2628	0.2766	0.034*	
H18B	1.6786	1.3522	0.3297	0.034*	
H18C	1.4648	1.3718	0.2844	0.034*	

### Atomic displacement parameters (Å^2^)

**Table:**

	*U*^11^	*U*^22^	*U*^33^	*U*^12^	*U*^13^	*U*^23^
Cl1	0.0219 (2)	0.0314 (2)	0.0211 (3)	−0.0074 (2)	−0.0005 (2)	−0.0049 (2)
Cl5	0.0219 (2)	0.0227 (2)	0.0234 (3)	−0.0029 (2)	−0.0021 (2)	−0.0059 (2)
C1	0.0151 (8)	0.0157 (9)	0.0215 (11)	0.0023 (8)	−0.0002 (8)	0.0001 (8)
C2	0.0214 (10)	0.0226 (10)	0.0193 (11)	0.0027 (9)	0.0009 (8)	0.0047 (8)
C3	0.0193 (9)	0.0232 (9)	0.0255 (11)	−0.0005 (9)	0.0070 (9)	0.0083 (8)
C4	0.0156 (9)	0.0156 (8)	0.0305 (12)	−0.0028 (8)	0.0000 (8)	0.0023 (8)
C5	0.0157 (9)	0.0123 (8)	0.0221 (11)	0.0024 (8)	−0.0027 (8)	0.0005 (7)
C6	0.0129 (8)	0.0116 (8)	0.0203 (10)	0.0030 (7)	0.0014 (7)	0.0009 (7)
C7	0.0188 (9)	0.0112 (8)	0.0150 (10)	0.0024 (7)	−0.0008 (8)	0.0005 (8)
C8	0.0163 (9)	0.0161 (9)	0.0182 (11)	0.0025 (7)	−0.0013 (8)	0.0034 (8)
C9	0.0160 (8)	0.0161 (9)	0.0195 (11)	0.0034 (7)	0.0020 (8)	−0.0002 (8)
O10	0.0188 (6)	0.0280 (7)	0.0176 (8)	−0.0035 (6)	−0.0014 (6)	0.0022 (6)
C11	0.0140 (8)	0.0150 (8)	0.0172 (10)	0.0021 (7)	0.0000 (8)	0.0005 (8)
C12	0.0180 (9)	0.0168 (9)	0.0146 (10)	0.0039 (8)	0.0026 (7)	0.0019 (7)
C13	0.0212 (9)	0.0170 (9)	0.0135 (10)	0.0022 (8)	−0.0034 (8)	−0.0031 (7)
C14	0.0169 (8)	0.0193 (9)	0.0149 (9)	0.0017 (9)	−0.0016 (8)	0.0009 (7)
C15	0.0170 (9)	0.0185 (9)	0.0163 (10)	0.0003 (8)	0.0022 (8)	0.0040 (8)
C16	0.0166 (9)	0.0121 (8)	0.0228 (12)	0.0005 (8)	−0.0013 (7)	0.0013 (8)
O17	0.0214 (7)	0.0264 (7)	0.0195 (8)	−0.0088 (6)	−0.0016 (6)	0.0005 (6)
C18	0.0202 (10)	0.0218 (9)	0.0256 (11)	−0.0072 (8)	0.0001 (8)	0.0037 (8)

### Geometric parameters (Å, °)

**Table:**

Cl1—C1	1.7484 (19)	C9—C11	1.494 (3)
Cl5—C5	1.747 (2)	C11—C14	1.396 (3)
C1—C2	1.392 (3)	C11—C12	1.409 (3)
C1—C6	1.410 (3)	C12—C13	1.384 (3)
C2—C3	1.387 (3)	C12—H12	0.95
C2—H2	0.95	C13—C16	1.397 (3)
C3—C4	1.389 (3)	C13—H13	0.95
C3—H3	0.95	C14—C15	1.391 (3)
C4—C5	1.388 (3)	C14—H14	0.95
C4—H4	0.95	C15—C16	1.395 (3)
C5—C6	1.410 (3)	C15—H15	0.95
C6—C7	1.473 (3)	C16—O17	1.368 (2)
C7—C8	1.342 (2)	O17—C18	1.442 (2)
C7—H7	0.95	C18—H18A	0.98
C8—C9	1.485 (3)	C18—H18B	0.98
C8—H8	0.95	C18—H18C	0.98
C9—O10	1.231 (2)		
			
C2—C1—C6	122.56 (18)	C8—C9—C11	118.26 (17)
C2—C1—Cl1	115.81 (16)	C14—C11—C12	118.64 (16)
C6—C1—Cl1	121.59 (15)	C14—C11—C9	118.51 (18)
C3—C2—C1	119.9 (2)	C12—C11—C9	122.73 (17)
C3—C2—H2	120	C13—C12—C11	120.46 (17)
C1—C2—H2	120	C13—C12—H12	119.8
C2—C3—C4	119.84 (18)	C11—C12—H12	119.8
C2—C3—H3	120.1	C12—C13—C16	120.05 (18)
C4—C3—H3	120.1	C12—C13—H13	120
C5—C4—C3	119.26 (17)	C16—C13—H13	120
C5—C4—H4	120.4	C15—C14—C11	121.28 (18)
C3—C4—H4	120.4	C15—C14—H14	119.4
C4—C5—C6	123.41 (19)	C11—C14—H14	119.4
C4—C5—Cl5	117.23 (14)	C14—C15—C16	119.28 (18)
C6—C5—Cl5	119.36 (15)	C14—C15—H15	120.4
C1—C6—C5	114.98 (18)	C16—C15—H15	120.4
C1—C6—C7	125.42 (17)	O17—C16—C15	123.72 (18)
C5—C6—C7	119.37 (18)	O17—C16—C13	116.00 (18)
C8—C7—C6	128.68 (19)	C15—C16—C13	120.27 (17)
C8—C7—H7	115.7	C16—O17—C18	117.23 (15)
C6—C7—H7	115.7	O17—C18—H18A	109.5
C7—C8—C9	119.76 (19)	O17—C18—H18B	109.5
C7—C8—H8	120.1	H18A—C18—H18B	109.5
C9—C8—H8	120.1	O17—C18—H18C	109.5
O10—C9—C8	121.29 (17)	H18A—C18—H18C	109.5
O10—C9—C11	120.44 (17)	H18B—C18—H18C	109.5
			
C6—C1—C2—C3	1.5 (3)	C7—C8—C9—C11	159.67 (16)
Cl1—C1—C2—C3	179.21 (14)	O10—C9—C11—C14	−12.1 (3)
C1—C2—C3—C4	−0.1 (3)	C8—C9—C11—C14	169.21 (16)
C2—C3—C4—C5	−1.3 (3)	O10—C9—C11—C12	163.91 (17)
C3—C4—C5—C6	1.4 (3)	C8—C9—C11—C12	−14.8 (2)
C3—C4—C5—Cl5	−179.22 (14)	C14—C11—C12—C13	−0.6 (2)
C2—C1—C6—C5	−1.4 (3)	C9—C11—C12—C13	−176.57 (16)
Cl1—C1—C6—C5	−178.94 (13)	C11—C12—C13—C16	−0.2 (3)
C2—C1—C6—C7	173.05 (17)	C12—C11—C14—C15	1.0 (2)
Cl1—C1—C6—C7	−4.5 (3)	C9—C11—C14—C15	177.10 (16)
C4—C5—C6—C1	−0.1 (2)	C11—C14—C15—C16	−0.5 (3)
Cl5—C5—C6—C1	−179.47 (13)	C14—C15—C16—O17	178.75 (16)
C4—C5—C6—C7	−174.87 (16)	C14—C15—C16—C13	−0.3 (3)
Cl5—C5—C6—C7	5.8 (2)	C12—C13—C16—O17	−178.48 (15)
C1—C6—C7—C8	35.1 (3)	C12—C13—C16—C15	0.6 (3)
C5—C6—C7—C8	−150.75 (19)	C15—C16—O17—C18	2.6 (2)
C6—C7—C8—C9	−175.13 (17)	C13—C16—O17—C18	−178.31 (16)
C7—C8—C9—O10	−19.0 (3)		

### Hydrogen-bond geometry (Å, °)

**Table:**

*D*—H···*A*	*D*—H	H···*A*	*D*···*A*	*D*—H···*A*
C4—H4···Cg1^i^	0.95	2.84	3.727	157
C7—H7···Cg2^i^	0.95	2.85	3.360	115

Symmetry codes: (i) *x*+1/2, −*y*−1/2, −*z*.
